# Supplementary material for: Assessment of genetic diversity and phylogenetic relationship of local coffee populations in southwestern Saudi Arabia using DNA barcoding
Source: PeerJ. 2023 Nov 24;11:e16486. doi: 10.7717/peerj.16486 (PMC10680449; doi:10.7717/peerj.16486)
Supplement: Table S1 [file peerj-11-16486-s001.docx]

**Table S1.** Singleton variable sites and parsimony information sites of the four chloroplasts barcode.

| **Gene** | **Parameter** | **Sites** | **Position** | **Total** | **Grand Total** |
| --- | --- | --- | --- | --- | --- |
| atpB-rbcl. | STVS | Two variants’ sites | 448 | 1 | 1 |
|  |  | Three variants’ sites |  | 0 |  |
|  | PIS | Two variants’ sites | 338 362 408 442 443 444 447 457 469 878 913 | 11 | 137 |
|  |  | Three variants’ sites | 335 339 340 350 356 360 361 363 364 366 369 370 383 387 388 394 395 396 398 399 400 403 404 405 410 414 416 417 430 432 433 434 435 437 438 439 441 445 446 451 452 453 454 455 459 460 464 465 466 467 470 471 488 489 490 494 495 496 497 498 499 500 505 507 510 512 513 515 520 522 523 524 530 531 534 536 539 540 543 545 546 549 552 553 554 555 562 563 564 567 568 569 570 595 602 766 768 771 776 779 780 784 789 794 795 826 828 829 834 844 845 855 866 867 869 874 882 896 897 907 908 911 914 915 921 924 | 126 |  |
| TrnL | STVS | Two variants’ sites | 40 60 103 134 149 155 165 173 178 182 186 192 215 216 218 219 237 242 246 335 343 344 369 378 386 390 391 397 398 400 402 405 407 408 411 413 417 418 419 427 430 431 | 42 | 52 |
|  |  | Three variants’ sites | 124 157 217 379 406 410 414 428 429 439 | 10 |  |
|  | PIS | Two variants’ sites | 37 42 49 50 58 63 64 71 72 73 74 76 77 79 84 85 86 87 88 92 93 99 100 102 105 106 108 109 112 113 114 116 117 123 126 129 130 131 132 140 141 142 146 148 154 156 161 162 163 167 168 170 172 180 184 190 191 195 196 198 199 200 201 203 204 221 223 226 227 228 232 233 239 244 245 248 250 251 252 253 255 256 258 260 261 263 266 267 270 273 274 275 276 278 280 281 282 284 288 289 294 295 297 302 303 304 305 309 312 313 314  315 318 320 321 322 323 324 327 328 329 330 332 333 334 337 341 342 346 347 348 349 351 352 353 354 356 357 358 360 361 362 363 365 367 373 375 382 387 392 393 401 426 437 440 | 155 | 182 |
|  |  | Three variants’ sites | 21 30 43 44 45 53 78 89 90 118 181 185 291 345 350 355 366 368 372 374 376 377 381 383 384 403 432 | 27 |  |
| **TrnL-TrnF** | STVS | Two variants’ sites | 32 33 50 51 64 91 224 256 268 271 273 281 282 284 285 286 287 290 291 293 297 300 301 307 308 310 312 314 315 318 320 321 324 325 327 328 330 332 336 337 338 340 346 347 348 349 350 351 353 354 356 357 362 363 364 365 366 367 369 370 371 373 374 375 376 385 388 389 392 393 396 403 406 407 410 411 416 419 426 427 429 430 432 443 444 445 447 448 452 455 456 457 459 460 467 470 471 472 475 476 484 487 490 494 495 497 501 504 505 506 508 510 520 521 535 543 548 549 551 | 119 | 133 |
|  |  | Three variants’ sites | 37 368 394 399 404 420 446 454 464 482 488 489 530 552 | 14 |  |
|  | PIS | Two variants’ sites | 31 46 47 86 88 382 386 400 414 415 422 423 431 442 449 450 453 458 463 473 474 477 478 479 485 486 492 493 502 518 525 533 538 541 547 | 35 | 45 |
|  |  | Three variants’ sites | 23 441 499 500 522 531 532 539 545 546 | 10 |  |

| TrnT-TrnL | STVS | Two variants’ sites | 33 35 36 37 39 40 41 42 43 45 46 49 51 52 53 60 62 63 65 69 73 76 77 78 80 81 82 84 86 87 88 91 92 96 97 98 99 101 106 107 108  109 114 115 122 124 125 126 127 129 130 131 132 136 137 138 141 142 143 150 151 152 154 155 161 162 163 166 167 169 174 175 177 178 180 184 189 190 191 192 195 196 197 202 204 211 213 217 221 222 223 224 227 229 230 233 236 237 249 253 254 256 257 258 259 260 261 262 265 267 268 270 271 273 275 276 283 284 287 288 294 299 301 303 304 305 306 310 311 312 313 321 322 326 327 328 330 332 335 339 346 347 351 352 354 356 357 358 359 366 368 371 375 377 379 381 382 385 386 391 394 398 400 403 405 411 414 415 416 417 420 422 428 432 435 437 445 446 447 449 451 456 457 464 468 471 473 475 476 477 481 482 490 491 492 493 497 498 499 500 502 504 509 513 514 515 516 519 520 527 528 529 532 533 534 535 538 542 543 544 545 546 548 551 554 565 568 576 579 580 582 595 598 599 600 601 604 605 618 626 630 631 633 638 639 640 642 643 645 646 647  649 652 654 655 656 661 664 668 670 674 676 678 679 680 682 684 685 688 691 695 696 697 698 701 703 706 709 711 714 715 716 718 719 722 723 725 727 749 752 756 760 762 766 768 771 774 777 778 782 791 800 814 | 303 | 338 |
| --- | --- | --- | --- | --- | --- |
|  |  | Three variants’ sites | 61 72 550 570 581 606 607 613 622 625 627 628 653 659 675 707 710 713 721 724 728 732 734 741 754 763 769 770 775 776 780 792 795 796 797 | 35 |  |
|  | **PIS** | Two variants’ sites | 28 240 393 421 444 452 470 530 541 547 549 573 574 586 590 592 594 608 614 619 620 621 624 636 650 660 665 666 671 677 683 705 708 712 731 748 751 753 755 798 799 802 805 812 819 820 821 | 47 | 74 |
|  |  | Four variants’ sites | 22 24 27 48 635 641 648 672 673 689 720 726 739 740 764 765 767 779 783 794 801 806 807 809 810 816 822 | 27 |  |
